# Trypanosoma-Cruzi Cross-Reactive Antibodies Longitudinal Follow-Up: A Prospective Observational Study in Hematopoietic Stem Cell Transplantation

**DOI:** 10.1371/journal.pone.0137240

**Published:** 2015-09-09

**Authors:** Esber S. Saba, Lucie Gueyffier, Marie-Laure Danjoy, Philippe Vanhems, Bruno Pozzetto, Mohamad Sobh, Hans Pottel, Mauricette Michallet, Maan A. Zrein

**Affiliations:** 1 INFYNITY-Biomarkers, Lyon, France; 2 Hospices Civils de Lyon, Lyon, France; 3 Laboratories of Bacteriology-Virology, GIMAP EA3064, Faculty of Medicine Jacques Lisfranc, Saint-Etienne, France; 4 Interdisciplinary Research Center, Catholic University Leuven, Kortrijk, Belgium; University of Pittsburgh, UNITED STATES

## Abstract

Antibodies named TcCRA “Trypanosoma cruzi Cross Reactive Antibodies” were detected in 47% of blood donors from French population unexposed to the parasite. In order to evaluate the passive or active transmissibility of TcCRA and further characterize its role and etiology, we have conducted a study in a cohort of 47 patients who underwent allogeneic Hematopoietic Stem Cell Transplantations (allo-HSCT). Donors and recipients were tested for TcCRA prior to transplantation. Recipients were further tested during follow-up after transplantation. Demographical, clinical and biological data were collected. Our primary end-point was to assess the risk of TcCRA acquisition after transplantation. During this initial analysis, we observed no seroconversion in patients receiving cells from TcCRA negative donors (n = 23) but detected seroconversion in 4 out of 24 patients who received hematopoietic stem cells from positive donors. Here, we are discussing possible scenarios to explain TcCRA-immune status in recipient after transplantation.

## Introduction

In the course of biomarker evaluation of a neglected disease (Chagas disease), we made a remarkable observation of a highly prevalent antibody specificity in unexposed European serum samples. These specific antibodies were named “*Trypanosoma cruzi* Cross Reactive Antibodies” (TcCRA) to stress out the fact that they were induced by another antigen than the one from *T*. *cruzi*, the causative agent of Chagas disease [1]. Focusing on opportunistic infection, we investigated TcCRA in patients undergoing allogeneic hematopoietic stem cells transplantation (allo-HSCT) and explored its potential impact on transplantation outcomes.

While allo-HSCT represents a curative therapy for some hematological malignancies and bone marrow failure states, relapse, graft-vs-host disease (GVHD) and infections continue to be major causes of morbidity and mortality following the engraftment [2–3]. We tested TcCRA in blood samples from 47 adult recipients who underwent allo-HSCT as well as samples from their donors. Under such conditions, serological antibody markers are typically observed either by immune reconstitution, by blood derivatives passive transfer or by an immune response to an infection. The main objective of the study was to identify possible TcCRA transmission or acquisition in allo-HSCT recipients.

## Patients and Methods

### Study design, Patient population

We performed a prospective observational study. Patients and donors, all from Caucasian ethnicity, were recruited between January and June 2011. This prospective analysis was approved by a Research institutional review board (CPP Sud-Est n° 2013/025) and all patients gave written informed consent and assent as appropriate. All patients who were eligible for allo-HSCT were included in the study. In this study, all blood samples were collected during patient’s standard care. Likewise, blood samples were drawn from the patients before starting the conditioning regimen, and then before hematopoietic stem cell infusion. During the early follow-up (FU), testing for TcCRA determination was performed once per week during the first 12 weeks following the transplantation. A later follow-up was performed for almost all patients at 3, 6, 9 and 12 months after transplantation. For donors, a blood sample was drawn at the time of hematopoietic stem cell harvest. Data concerning age, sex, stem cell source, conditioning regimen, complications and infections after transplantation and follow-up were collected from the allo-HSCT registry of the hematological department of Lyon.

### Serological testing

TcCRA were measured in serum samples using an in-house ELISA test. The used method and reagents are described in SABA *et* al 2013 [1]. All the collected samples were tested in duplicate at least one time, if necessary twice, for validation. For some patients we tested serum for anti-measles, anti-mumps and anti-CMV IgGs. Those tests were performed by using the corresponding Enzygnost kit from SIEMENS.

### Conditioning regimen and GVHD prophylaxis

The conventional conditioning regimen was mainly a combination of cyclophosphamide and total body irradiation (TBI). The reduced-intensity conditioning regimen was mainly fludarabine combined with melphalan, cyclophosphamide, TBI and busulfan. The standard GVHD prophylaxis after the transplantation consisted of cyclosporine A and methotrexate. Steroids and/or Cyclosporine were used for the treatment of established acute or chronic GVHD.

### Viral monitoring

Patient’s serological status of cytomegalovirus (CMV), Epstein-Barr virus (EBV), herpes simplex virus (HSV), varicella zoster virus and toxoplasmosis were determined prior to transplantation. All HSCT patients were tested by quantitative real-time PCR for EBV, CMV and HHV-6 during the FU after transplantation. All patients received herpes prophylaxis *i*.*v*. valacyclovir. CMV reactivation was treated with valgancyclovir and EBV reactivation with rituximab if testing confirmed the viral activation.

### Statistical analysis

Statistical analysis was done using the SPSS ver.19 and Graph-pad prism 5. The two-tailed *P* value was considered significant when <0.05. Mann-Whitney and Fisher exact tests were used to calculate significance of continuous and categorical variables respectively.

## Results

### Patients’ characteristics

Forty seven recipients and their donors were included in the study. Among them there were 26 males and 21 females with a median age of 51 years (range: 35–58). TcCRA antibody were followed during a median of 280 days. Diagnosis before transplantation was acute lymphoblastic and myeloid leukemia (n = 19, n = 7), myelodisplesia (n = 7), non-Hodgkin’s lymphoma (n = 6) and other diagnosis including Hodgkin’s lymphoma (n = 1), Myeloproliferative syndrome (n = 2), solid tumor (n = 1) and aplasia (n = 4). As HSC source, 22 patients have received peripheral blood cells, 23 bone marrow and 2 cord blood cells from 32 unrelated donors, HLA matched (n = 18) and HLA-mismatched (n = 14) and 15 siblings donors. For ABO compatibility, 18 patients were compatible, 13 had minor incompatibility and 16 had major incompatibility with their respective donor. As for conditioning regimens, 23 patients had a myeloablative and the remaining 24 patients had a reduced intensity conditioning. Twenty patients died at different time points during the FU, 15 from transplantation related complications and 5 from disease recurrence. Consequently, the numbers of available samples at 3, 6, 9 and 12 months were respectively 41, 39, 31 and 27. The population was divided in two groups according to the donors’ TcCRA status, all characteristics are shown in Table 1.

**Table 1:** Patients’ characteristics.

		Group donor TcCRA (-)	Group donor TcCRA (+)
**Recipient’s TcCRA BL (OD)**		**0.55 (0.29–0.81)**	**0.41 (0.20–0.64)**
**Recipient’s Age (years)**		**52 (35–57)**	**50 (38–58)**
**FU (days)**		**304 (152–367)**	**277 (104–368)**
**Total nb of cells in the graft (10^8 cells/Kg)**		**3.1 (1.6–9.8)**	**4.8 (2.3–13.1)**
**CD 34+ (10^6 cells/kg)**		**3.05 (1.1-.7)**	**4.5 (2.2–6)**
**CD 3+ (10^6 cells/kg)**		**39.6 (17.3–249)**	**51.8 (21.6–206)**
**Recipient’s gender (%)**	**Female**	**11 (50)**	**11 (50)**
	**Male**	**12 (48)**	**13 (52)**
**Deceased (%)**	**No**	**13 (48)**	**14 (52)**
	**Yes**	**10 (50)**	**10 (50)**
**HLA**	**Match**	**13 (39)**	**20 (61)**
	**Mismatch**	**10 (71)**	**4 (29)**
**Conditioning regimen (%)**	**MA**	**13 (57)**	**10 (53)**
	**RIC**	**10 (43)**	**13 (57)**
**ABO system**	**Compatible**	**9 (50)**	**9 (50)**
	**Minor incompatibility**	**8 (62)**	**5 (38)**
	**Major incompatibility**	**7 (44)**	**9 (56)**
**Stem cells source (%)**	**PB**	**11 (50)**	**11 (50)**
	**BM**	**10 (44)**	**13 (56)**
	**UC**	**2 (100)**	**0 (0)**
**Relation donor- recipient (%)**	**Sibling**	**8 (53)**	**7 (47)**
	**Unrelated**	**15 (47)**	**17 (53)**
**CMV serology recipient (%)**	**IgG (-)**	**8 (57)**	**6 (43)**
	**IgG (+)**	**15 (46)**	**18 (54)**
**EBV serology recipient (%)**	**IgG (-)**	**0**	**0**
	**IgG (+)**	**23 (48)**	**24 (50)**
**HSV1 serology recipient (%)**	**IgG (-)**	**3 (43)**	**4 (57)**
	**IgG (+)**	**20 (50)**	**20 (50)**
**VZV serology recipient (%)**	**IgG (-)**	**1**	**0**
	**IgG (+)**	**22 (48)**	**24 (52)**
**Toxoplasmosis serology recipient (%)**	**IgG (-)**	**9 (69)**	**4 (31)**
	**IgG (+)**	**14 (41)**	**20 (59)**

Abbreviations: TcCRA = *Trypanososma cruzi* Cross Reactive Antibodies; HLA = Human Leucocyte Antigen; FU = Follow-up; BL = baseline; CMV = cytomegalovirus; EBV = Epstein Barr virus; HSV1 = herpes simplex virus 1; VZV = varicella zoster virus; MA = myelo-ablative; RIC = reduced intensity conditioning; PB = peripheral blood; BM = bone marrow; UC = umbilical cord.

**Fig 1:**
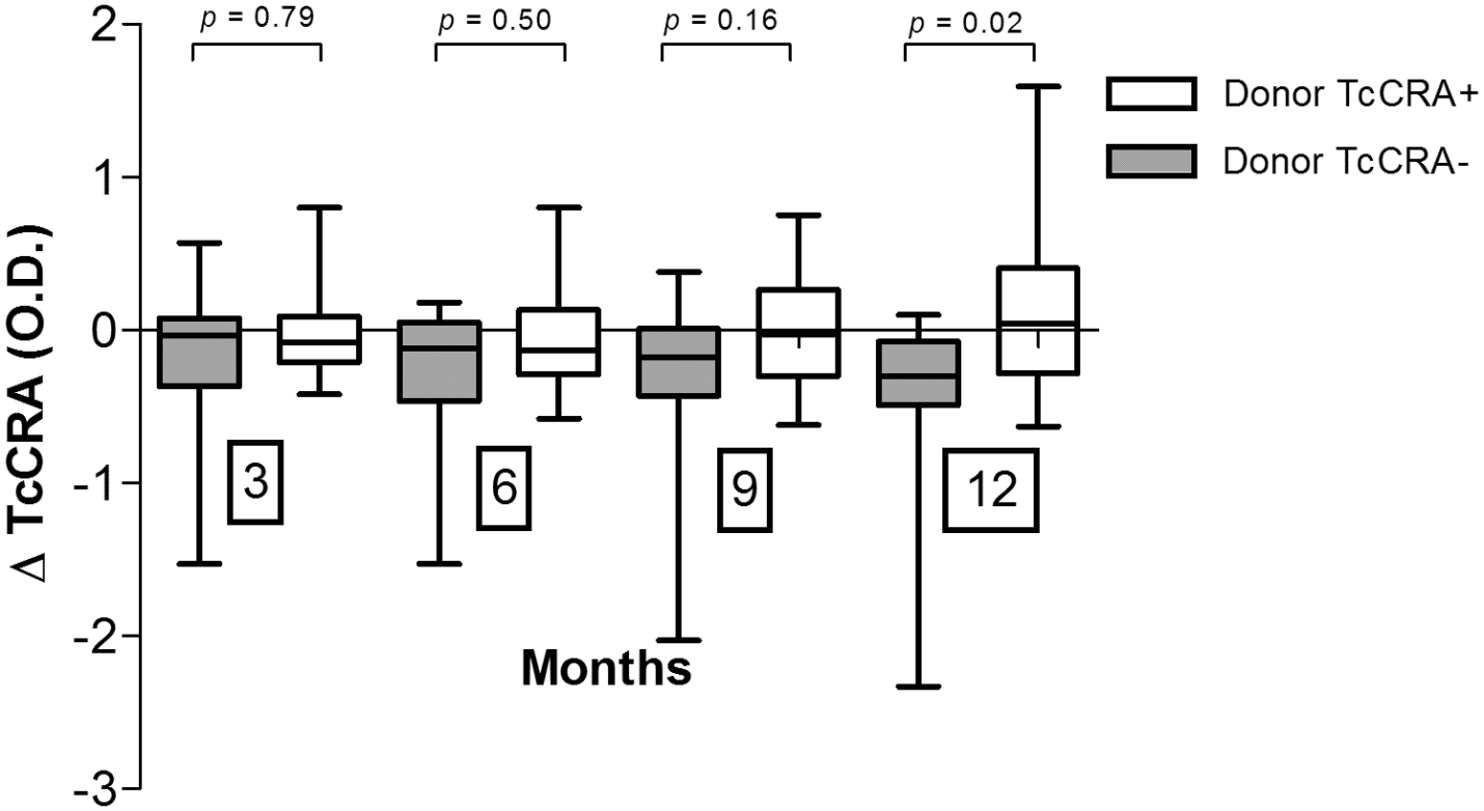
Effect of donor’s TcCRA serological status on patients’ TcCRA-signal evolution. ΔTcCRA was calculated at 3, 6, 9 and 12 months after graft to evaluate the gain or loss in TcCRA signal. The numbers of available samples at 3, 6, 9 and 12 months were respectively 41, 39, 31 and 27.

To monitor TcCRA marker, we calculated the difference between the signals at baseline (BL) and those measured at 3, 6, 9 and 12 months after transplantation (ΔTcCRA). Then we compared the distribution of these values between the two groups (Fig 1). A difference between groups became measurable at 9 months and significant at 12 months after the transplantation. ΔTcCRA values showed a significant decrease in patients receiving cells from a seronegative donor as compared to an increase in patients receiving cells from a seropositive donor.

### Longitudinal follow-up of TcCRA over one year after transplantation

**Fig 2:**
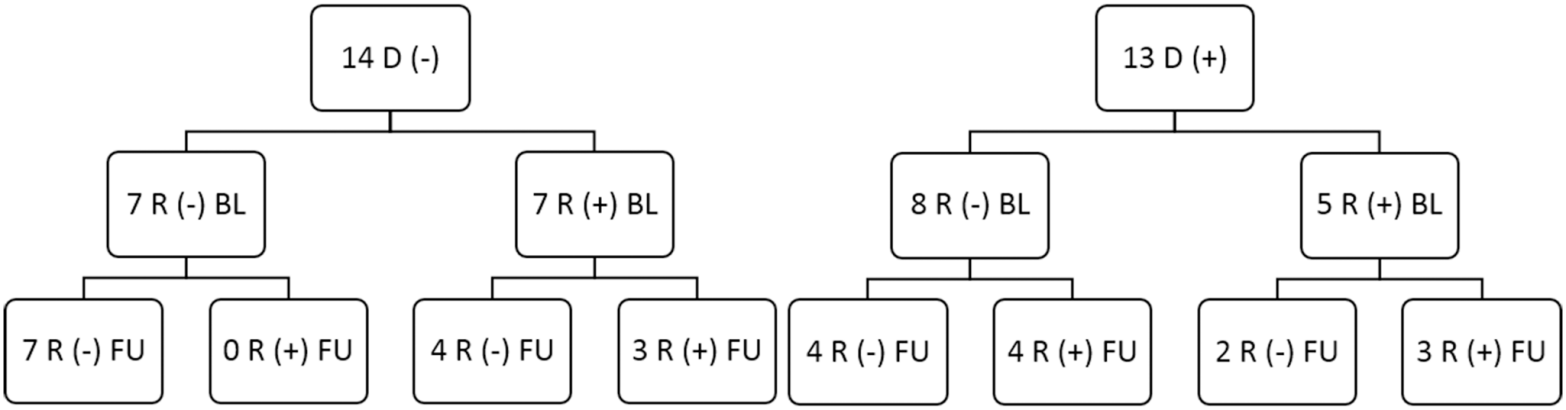
Classification of patients who survived more than 9 months after the transplantation at baseline (BL) and at Follow up (FU) according to their donor’s TcCRA serological status.

Based on the observation of a late influence of the donor’s TcCRA-status, we focused our analysis on the 27 patients with available samples more than 9 months after transplantation (Fig 2).

**Fig 3:**
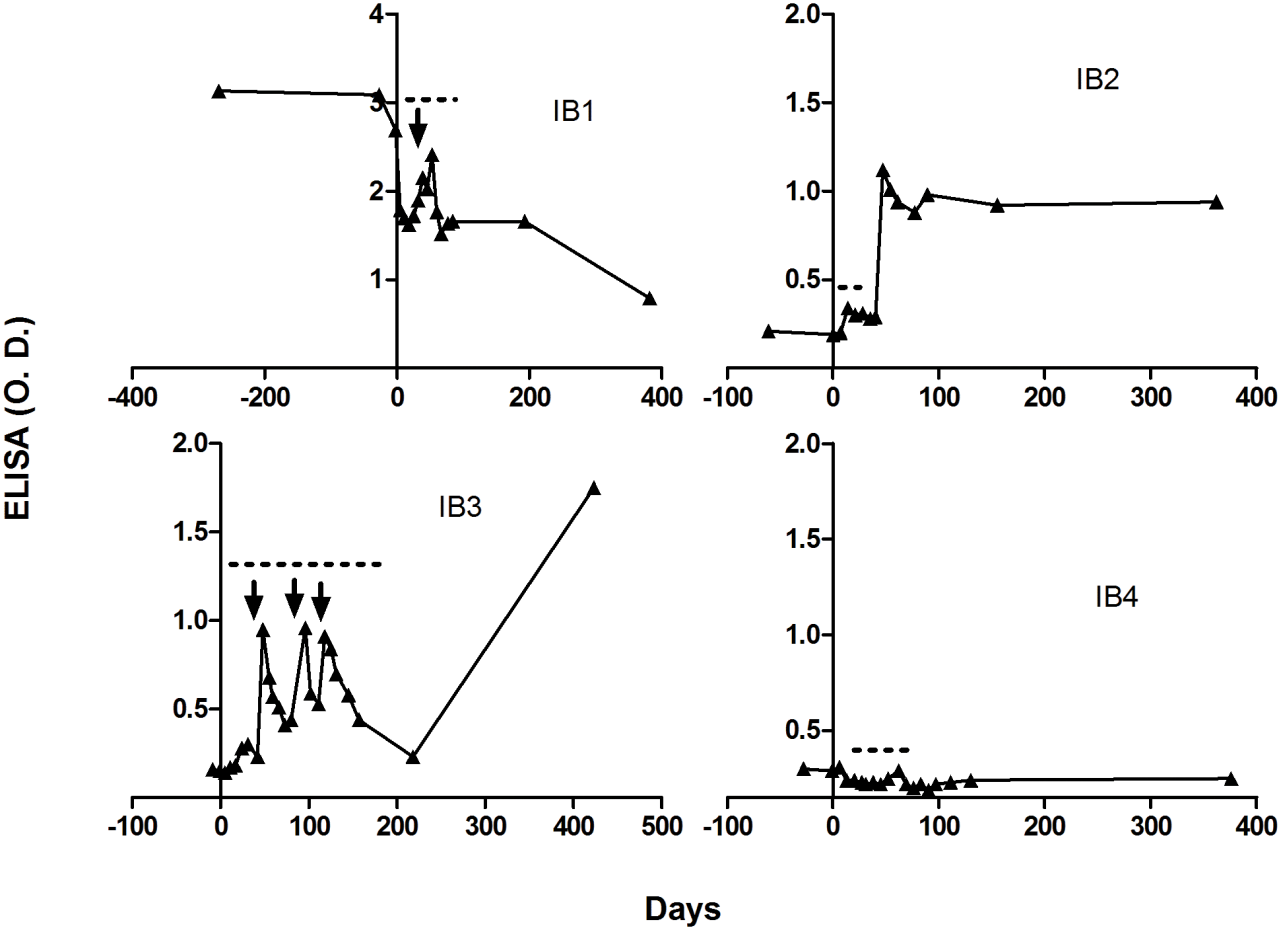
TcCRA profile of four patients during the follow-up. The X axis represents time in days, where day zero designates the day of engraftment. The Y axis represents TcCRA signals (O.D.) in recipients. Black arrows symbolize the dates of *i*.*v*.Ig injections while dotted black lines indicate transfusion periods of other blood-products.

We established a graphical representation of TcCRA signal evolution for each of these 27 patients and observed 4 different profiles depicted as IB1, IB2, IB3 and IB4 in Fig 3: The first pattern is characterized by a general downward trend of TcCRA signals, in 8 patients with high level of TcCRA (> 1 OD) at inclusion, a decrease in signal was observed after the conditioning phase until the end of the follow-up (Fig 3, IB1). The second one, documented only in one patient, TcCRA signal rises early after transplantation (47 days) and then stabilized until the end of the follow-up period. The third profile is common for three patients, shows a late increase in TcCRA signal, happening 200 days after transplantation. The fourth profile shows no TcCRA variation in 15 patients (Fig 3, IB4). We have also observed transient peak of TcCRA signal, we were able to correlate these peaks with a passive transfer of antibodies, carried out mainly through i.v.Ig (intravenous immunoglobulin) injections (symbolized by black arrows in Fig 3) and to a lesser extent through platelet and red blood cell transfusions.

### Influence of the donor’s serological status on the recipient’s post-graft antibody repertoire

**Fig 4:**
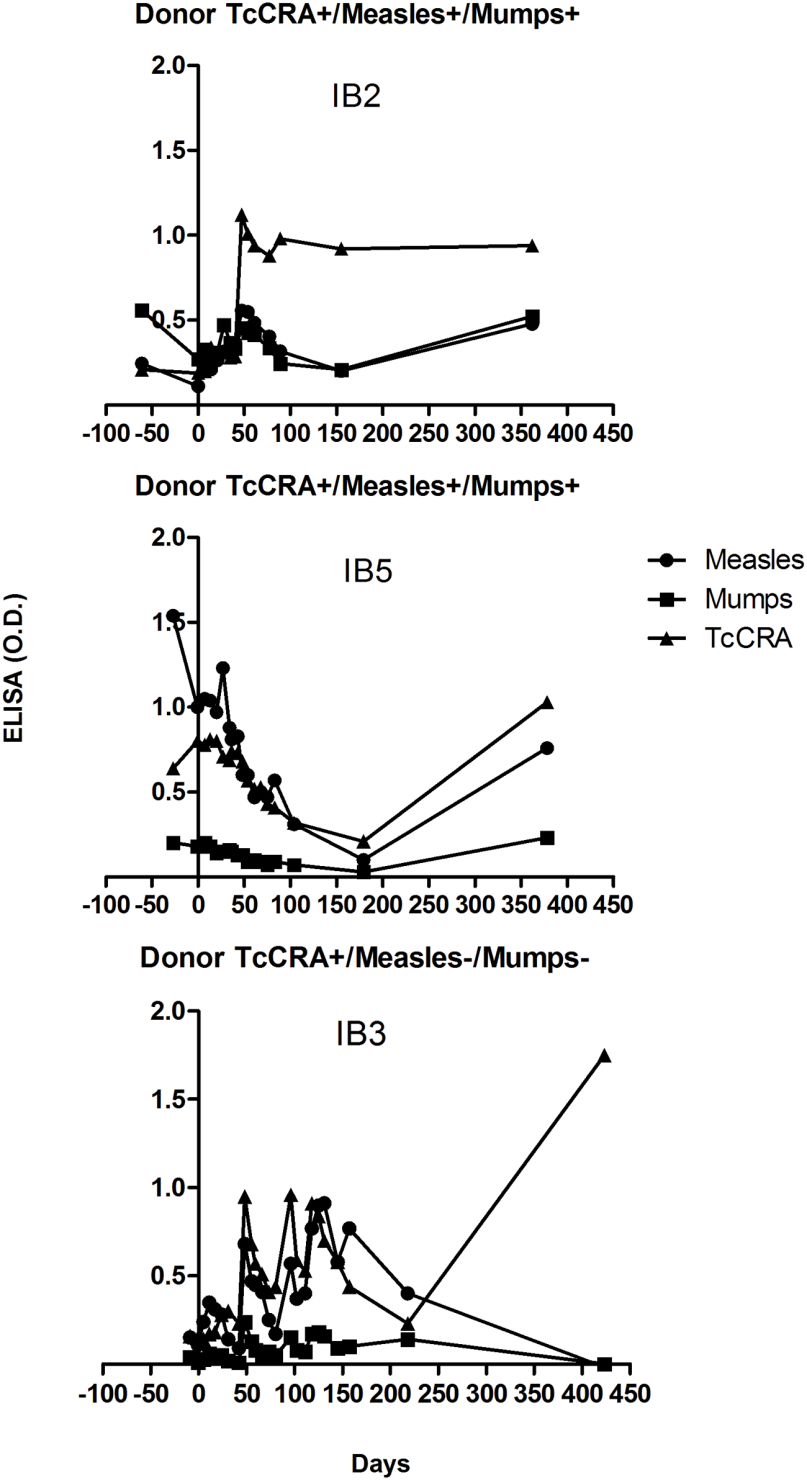
Serology profiles of three different patients including TcCRA, IgG anti-measles virus and IgG anti-mumps virus. Donor’s serological status is presented for each patient as well. 0.2 (O.D.) represents the cutoff for anti-measles and anti-mumps antibodies.

Interestingly, three patients with a late increase in TcCRA signals have had a TcCRA seropositive donor. This observation seems to be related with immune reconstitution after transplantation. To verify the hypothesis of a late immune reconstitution is of donors’ origin, we tested patients for anti-measles and anti-mumps IgGs which are representative of infections widely prevalent but unlikely to reactivate. We found that, over one year of follow-up, patients were more likely to acquire their donor’s immune repertoire, as was apparent for patients IB5 and IB3 (Fig 4). In contrast, patient IB2 showed an early conversion of TcCRA status that doesn’t seem related to immune reconstitution as was shown for Measles and Mumps antibodies.

### Early post-graft specific antibody forming ability

**Fig 5:**
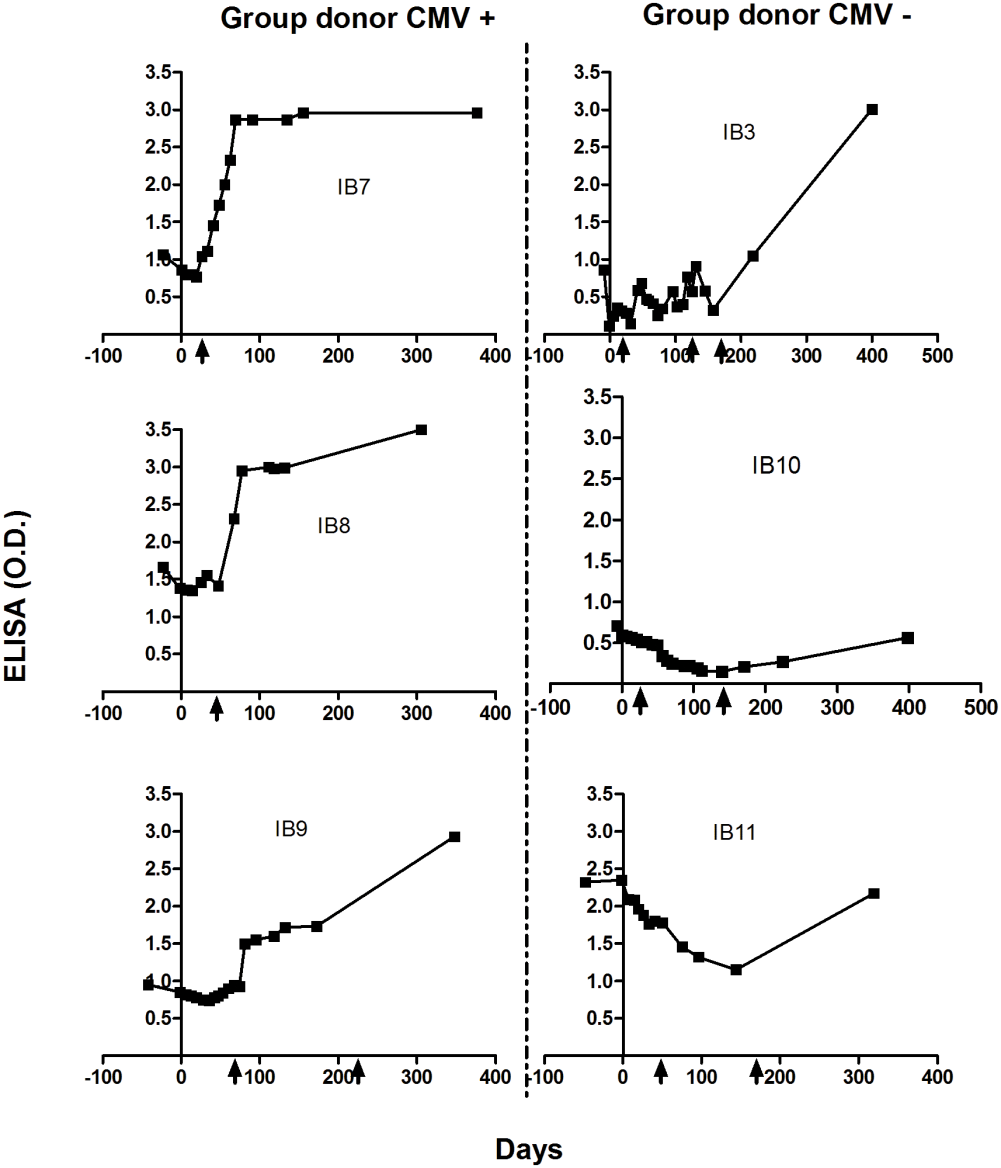
Patients’ antibodies anti-CMV profiling. The black arrows indicate the day of post-graft CMV reactivation (>100 copies/ml measured by PCR).

To further investigate the observation documented for the patient IB2, with an early increase in TcCRA signal, we selected a third infectious agent (CMV) as a comparison basis, also largely spread and strongly susceptible to reactivation in allo-HSCT patients. We measured anti-CMV antibodies in recipients who underwent CMV reactivation (Fig 5). All tested patients were CMV positive at inclusion. We found that only Patients who received cells from a CMV seropositive donor were able to specifically respond upon CMV reactivation. Fig 5 showed that these patients had an increase in anti-CMV antibodies after the detection of CMV viremia (symbolized by a black arrows) occurring within the first three months after transplantation. However, this was not observed in the case of patients with a CMV seronegative donor who were not able to respond promptly after the reactivation and failed to produce specific anti-CMV antibodies during the early post-transplantation periods.

## Discussion

TcCRA are newly discovered antibodies of unknown origin, which might be the result of infection with an asymptomatic latent agent [1]. In the present study, we prospectively evaluated the expression and dynamics of TcCRA in 47 pairs of adult HSCT donors/recipients. TcCRA expression was shown to be stable over time in patients who had samples before transplantation (Fig 3, IB1). The loss or decline of immunity is common in the early phase following allo-HSCT and generally contributes to patients’ susceptibility to infections [4]. This decline has been well documented for immunity acquired in childhood through natural infections or vaccination against mumps, rubella and poliomyelitis for example [5]. In our study, we found that TcCRA-positive patients experienced a similar trend with at least 50% loss of reactivity 90 days after transplantation (Data not shown). We also observed that transient peaks in TcCRA signals mainly correlate with exogenous immunoglobulins (IgGs) administration. The level of passively transferred IgGs progressively decreased until complete clearance 30–60 days after the initial injection. Taking into consideration that TcCRA is present in 47% of blood donations, we were not surprised to detect its presence in human blood derivatives such as polyclonal therapeutic immnunoglobulins.

Our primary end-point was to assess the risk of TcCRA-immunity acquisition after the recipient exposition to either a positive or a negative donor following allo-HSCT. We are reporting here a TcCRA immunity acquisition in four patients after engraftment of cells from positive donors, clearly unrelated to IgGs given intravenously. In 3/4 patients, TcCRA acquisition showed a similar profile to the acquisition of anti-measles and anti-mumps antibodies. The antibody production observed in these patients beyond 6 month after allo-HSCT appeared to reflect the transfer of immunity from donors’ cells after complete hematopoietic reconstitution [6]. This hypothesis is built on four pieces of evidence: “1” the antibodies that were induced in recipients after allo-HSCT matched the donors’ immune repertoire, “2” when donors were negative, recipients were found to be negative, including recipients who were positive at base-line, “3” recipients who developed serum IgG titers to measles and mumps were unlikely to be “environmentally re-immunized”, “4” passive antibodies transferred through administered blood derivatives did not influence antibody titers 6 months after transplantation. To our view point, evidences mentioned above were not sufficient to explain why the TcCRA profile for patient IB2 was different (Fig 3). Interestingly, in this case, the donor TcCRA signal was the highest amongst all donors. The early development and the consequent persistence of this immune response were particularly remarkable. The levels of detected antibodies suggested a main role of specific B cells activation upon specific stimulus. To challenge this idea, we measured anti-CMV antibodies in patients who underwent CMV reactivation. Remarkably, only recipients with CMV seropositive donors were capable to respond quickly after reactivation. It doesn’t seems plausible that the detected anti-CMV antibodies resulted from the persistence of differentiated host B cells, because it would also be observed in patients receiving cells from CMV-seronegative donors (Fig 4). It was evident that anti-CMV antibodies response was detected before complete hematopoietic engraftment which usually takes from 6 months up to 2 years [7–8]. Therefore, the adaptive transfer of primed immune cells from the donor to the recipient appeared to be a more suitable scenario. Actually, the adaptive transfer of immunocompetent cells routinely occurs during allo-HSCT [9]. This was reported in publications on the effectiveness of vaccination after HSCT [5–10], as well as through cancer therapy where immunized donor against tumor specific antigen might increase the recipient anti-cancer response after HSCT [11–12].

The early rise in TcCRA signal in patient IB2 suggested a possible stimulation of donor’s primed B-cells by the infectious agent inducing TcCRA. Being TcCRA-seronegative at inclusion, it was unlikely that the agent was present in the host before allo-HSCT. It could therefore derive either concomitantly with the graft or from an external source like transfusion or environmental exposure. The last possibility was however unlikely to explain the seroconversion in patient IB2, because of its occurrence at the period of time after transplantation when patients were carefully isolated from the outside world. Moreover, the risk of transfusion related infection was highly minimized by the use of leuco-reduced products [13]. Given the high TcCRA level of the donor and the fact that latent viruses are frequently transmitted via the transplanted cells [14], we couldn’t unacknowledged the possibility of agent transmission via donor cells, at least for this specific case.

In conclusion, this study has undoubtedly put forward our hypothesis of the implication of an unknown, probably latent, asymptomatic virus responsible of TcCRA induction transmitted possibly by blood cell. This agent may be part of the uncharacterized viruses that constitute 15% of healthy individuals’ blood virome as it was proposed by Popgeorgiev N *et al*. in their work on Marseillevirus [15–16]. Our data showed the need to pursue research on the composition of blood derived products used in allo-HSCT setting that may have a clinical significance.
